# Structural predictors of response to intra-articular steroid injection in symptomatic knee osteoarthritis

**DOI:** 10.1186/s13075-017-1292-2

**Published:** 2017-05-08

**Authors:** Nasimah Maricar, Matthew J. Parkes, Michael J. Callaghan, Charles E. Hutchinson, Andrew D. Gait, Richard Hodgson, David T. Felson, Terence W. O’Neill

**Affiliations:** 1 Arthritis Research UK Centre for Epidemiology, Division of Musculoskeletal and Dermatological Sciences, Faculty of Biology, Medicine and Health, Manchester Academic Health Science Centre, University of Manchester, Manchester, UK; 2 NIHR Manchester Musculoskeletal Biomedical Research Unit, Central Manchester University Hospitals NHS Foundation Trust, Manchester Academic Health Sciences Centre, Manchester, UK; 3 Department of Physiotherapy, Salford Royal Hospital NHS Foundation Trust, Manchester, UK; 4 Department of Health Professions, Manchester Metropolitan University, Manchester, UK; 5 Imaging Sciences, University of Warwick, Warwick Medical School, Coventry, UK; 6 Centre for Imaging Sciences, Institute of Population Health, The University of Manchester, Manchester, UK; 7 Clinical Epidemiology Unit, Boston University School of Medicine, Boston, MA USA; 8 Department of Rheumatology, Salford Royal Hospital NHS Foundation Trust, Manchester, UK

**Keywords:** Knee osteoarthritis, Clinical trial, Intra-articular steroid injection, Predictors of response

## Abstract

**Background:**

The aim was to examine if structural factors could affect response to intra-articular steroid injections (IASI) in knee osteoarthritis (OA).

**Method:**

Persons with painful knee OA participated in an open-label trial of IASI where radiographic joint space narrowing (JSN) and Kellgren-Lawrence (KL) grade, whole-organ magnetic resonance imaging (MRI) scores (WORMS) and quantitative assessment of synovial tissue volume (STV) were assessed on baseline images. Participants completed the Knee Injury and Osteoarthritis Outcome Score (KOOS) and a question about knee pain with a visual analogue scale for pain during nominated activity (VAS_NA_), and Outcome Measures in Rheumatology (OMERACT)-Osteoarthritis Research Society International (OARSI) criteria were used to assess responder status within 2 weeks (short term) and 6 months (longer term). Regression models were used to examine predictors of short and longer term response to IASI.

**Results:**

Subjects (*n* = 207) attended and had IASI. Information on responder status was available on 199 participants. Of these, 188 subjects, mean age 63.2 years (standard deviation (SD) 10.3), 97 (51.6%) female, had x-rays and 120 had MRI scans available. Based on the OMERACT-OARSI criteria, 146 (73.4%) participants responded to therapy and 40 (20.1%) were longer term responders. A few factors were associated with a reduced KOOS-pain and VAS_NA_ response though none were associated with OMERACT-OARSI responder status in the short term. Higher MRI meniscal damage (odds ratio (OR) = 0.74; 95% CI 0.55 to 0.98), increasing KL maximal grade (OR = 0.43; 95% CI 0.23 to 0.82) and joint space narrowing (JSN) maximal score (OR = 0.60; 95% CI 0.36 to 0.99) were each associated with a lower odds of longer term responder status. Baseline synovitis was not associated with treatment response. The predicted probability of longer term response decreased from 38% to 12% as baseline maximal JSN increased from grade 0 to 3.

**Conclusion:**

Compared with those who have mild structural damage, persons with more severe knee damage on either MRI or x-ray are less likely to respond to knee IASI.

**Trial registration:**

ISRCTN.com, ISRCTN07329370. Registered 21 May 2010. Retrospectively registered

## Background

Intra-articular steroid injection (IASI) is an effective [1], widely used [2] and recommended treatment [3] for individuals with symptomatic osteoarthritis (OA) of the knee with short term pain relief lasting up to 3–4 weeks compared with placebo injection [1, 4–6], with some persons having longer term response up to 24 weeks [4, 7]. There are, however, significant variations in both the magnitude and duration of symptom relief following steroid injections [1, 4, 5, 8]. Such variation may be due to differences in the phenotypic expression, including severity of the disease; however, there are few published data on this issue and those studies that have examined structural factors predicting response have been small [6, 7, 9–14] and findings have been discrepant [6–8, 11, 13, 15]. Further, only radiographic [7, 9, 13, 15] and arthroscopic [7] predictor factors have been studied. Also in one trial factors predictive of IASIs were studied after combined treatment with arthroscopic lavage [7].

In our recent open-label study of IASI in knee OA [16], not all participants responded to the therapy in the short term (within 2 weeks). Of those who responded in the short term the majority, though not all, had a recurrence of pain within 6 months. As part of this study, we examined whether factors representing structural features of OA or of inflammation might identify persons who were likely to respond to IASI either in the short or longer term. Our study of IASI predictor of response was larger in scale and longer in follow up than prior studies, and also involved scoring of individual features through contrast-enhanced (CE)-magnetic resonance imaging (MRI), offering the opportunity to identify other OA features and synovial factors affecting response to IASI treatment. To optimize selection of persons for IASI treatment, it would be helpful to separate responders from non-responders.

We hypothesised that those with more severe joint damage would be less likely to respond, while those with an underlying “inflammatory” phenotype characterised by synovitis/effusion would be more likely to respond. Using data from our study, we looked at the impact of these “structural” factors based on radiographic and MRI on response to therapy in the short and longer term.

## Methods

### Participants

Men and women (*n* = 209) aged 40 years and over were recruited from primary and secondary care clinics for participation in an open-label study examining the efficacy of IASI in symptomatic knee OA (ISRCTN07329370). Subjects were included if they reported moderate knee pain for more than 48 hours in the previous 2 weeks on global rating or scored more than 7 out of 32 on the Knee Injury and Osteoarthritis Outcome Score (KOOS) [17], for questions P2–P9 (question P1 relates to frequency of knee pain, which is irrelevant given the inclusion criteria on pain frequency). Inclusion criteria included imaging confirmation of OA either radiologically in the index knee (in any compartment on anteroposterior (AP), skyline or lateral projection knee radiographs obtained within the previous 2 years) with a Kellgren-Lawrence (KL) score of 2 or more or, on MRI scan or at arthroscopy. For MRI and arthroscopy, we required typical changes of OA with at least evidence of cartilage loss. Exclusion criteria included gout, septic arthritis, inflammatory arthritis, hyaluronic acid or steroid injection within the previous 3 months, knee surgery within the previous 6 months, concurrent life threatening illnesses and any contraindication to MRI scanning.

### Screening and baseline assessment

Subjects were assessed for eligibility at a screening visit [16]. For those in whom previous knee imaging within the previous 2 years was not available, radiographs of the index knee were performed at the screening assessment. Those who fulfilled the inclusion/exclusion criteria were invited to attend baseline assessments. Subjects completed a series of questionnaires including the KOOS-pain scale [17], a global Likert scale and visual analogue scale (VAS) for pain during an activity that a patient nominated as being most troublesome (VAS_NA_). For those subjects who took part in the MRI sub-study, the MRI scans were undertaken immediately prior to IASI. Following the MRI scan the joint was injected with 80 mg methylprednisolone acetate (without local anaesthetic) with aspiration of synovial fluid in cases of clinical effusion. We treated and studied one knee per participant.

### Follow up

Short term and longer term response was assessed using the Outcome Measures in Rheumatology-Osteoarthritis Research Society International (OMERACT-OARSI) responder criteria based on the KOOS-pain scale and global Likert scale [18]. A responder was defined as either (1) greater or equal to 20% change in KOOS-pain score and a “slightly” or “much better” score on the 5-point Likert scale for change in pain, or (2) greater or equal to 50% change in the KOOS-pain score; in both cases an absolute change of at least 3 units if the baseline KOOS score was 15 or less. Participants were seen usually within 2 weeks after the injection and we characterised their response then as short term response. Based on these criteria those who had not responded were not further followed up. Those who responded were followed with regular telephone calls during which the same KOOS-pain questions and global Likert scale were administered. Those whose pain rebounded to within 20% of the baseline KOOS-pain score were defined as having relapsed and were seen again for a final follow up. Those whose pain levels did not return to this level at 6 months of follow up were classified as “longer term responders”.

### Magnetic resonance imaging: acquisition and analysis

Using a 3-T Philips MRI scanner, sagittal post-contrast T1W FS (repetition time (TR) 550 ms, echo time (TE) 20 ms, field of view (FOV) 14 cm × 14 cm, size 320 × 320) and sagittal pre-contrast 3-D WATSC (TR 20 ms, TE 4.7 ms, FOV 15 cm × 15 cm, size 288 × 288) scans were obtained. Semi-quantitative assessment of the MRI images over 14 areas using the Whole-Organ Magnetic Resonance Imaging Score (WORMS) was used by an experienced musculoskeletal (MSK) radiologist to grade effusion, cartilage, bone attrition, bone marrow lesion (BML), synovitis, osteophytes, bone cyst, menisci and ligaments within the knee, using an approach previously described in which higher scores indicate greater severity of the feature [19]. Synovitis was scored 0–1–2–3 according to increasing synovial thickness [19]. Bone attrition, BML, effusion and cyst were scored 0–3, cartilage and menisci were scored 0–6 and osteophytes were scored 0–7 in each of the 14 areas [20]. For all MRI features, we used the maximal score, that is, the worst scoring in any region of the knee. Synovitis and effusion were scored using the sagittal contrast-enhanced (CE) MRI sequence [19, 20]. Intra-reader reliability was assessed by the same reader re-evaluating 19 films after an interval period, with weighted kappa scores of 0.71, 0.70, 0.63, 0.77, 0.88, 0.77 and 0.91 for cartilage, cysts, osteophytes, BMLs, menisci, synovitis and effusion, respectively. The CE images were then assessed quantitatively for synovial tissue volume (STV) [16]. To evaluate repeatability of assessment of synovial volumes, the segmenter was asked to segment 10 knees randomly selected (without replacement) from those that had been previously segmented with new identification numbers assigned [16]. The intra-class correlation coefficient (ICC) for intra-reader reliability of the manual segmentation was 0.94.

### Radiographs

Knee radiographs were evaluated by an experienced MSK radiologist (CEH) who scored the KL and joint space narrowing (JSN) grades on skyline, weight-bearing AP and lateral projection radiographs of all participants for whom films were available. The KL and JSN grades were scored separately with KL scores 0–4 to denote the increasing level of joint degeneration [21], while JSN 0–3 indicated none, mild, moderate and severe reduction in joint space [22]. We looked also at anatomic mal-alignment. This was assessed on weight-bearing AP radiographs by one assessor (NM) using software installed within the hospital imaging system by calculating the angle from the intersection of the line from the midpoint of tibial spines bisecting the femoral shaft with the line from the midpoint of the tibial spines bisecting the tibial shaft [23]. The ICC was 0.99 for intra-rater reliability in the measurement of knee mal-alignment angle performed 3 months apart through random generated numbers of 50 images.

### Statistical analysis

Subject characteristics were summarised using means and standard deviations (SD) for normally distributed variables and medians and interquartile ranges (IQR) for variables with a skewed distribution. We used the paired *t* test to look for change in pain, assessed using the KOOS and VAS_NA_ between baseline and the post-injection visit. We used a fixed-effects panel regression model to examine whether there was any association between the baseline structural factors and change in pain, a continuous outcome variable (assessed using both KOOS and VAS_NA_) between baseline and initial follow up. For effusion, cartilage, bone attrition, BML, synovitis, osteophytes, bone cyst, menisci and ligaments we focused our analyses on the maximal WORMS for that feature and we included it in the model as a continuous variable; results were expressed as unstandardized b-coefficients and 95% confidence interval (CI). Logistic regression was used to determine whether baseline imaging parameters were associated with both short term and also longer term responder status (yes/no as outcome) to therapy. Results were expressed as odds ratios (OR) and 95% CI. Each factor of interest was investigated separately using a bivariate model, with either short term or longer term responder status as the outcome, and the factor of interest as the predictor variable. Data for short term and longer term response were analysed using a maximal case analysis using all available data for all variables. To examine the predictive probability of responder status (short/longer term), we extracted the predictive probabilities using the logistic regression model. Statistical analyses were undertaken using Stata version 13.1 (StataCorp, College Station, TX, USA).

## Results

### Participants

**Fig. 1:**
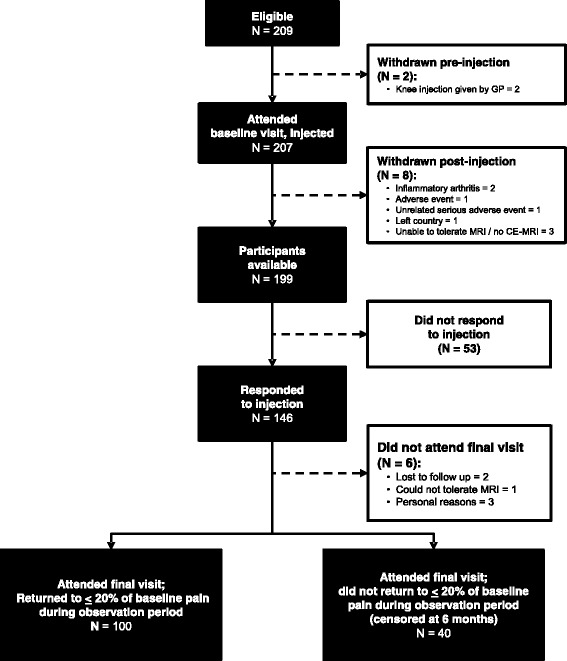
Preferred reporting items for systematic reviews and meta-analyses (*PRISMA*) flow chart of participants for assessments of radiographs and magnetic resonance imaging (*MRI*) scans. *GP* General Practitioner, *CE* contrast-enhanced

We recruited 209 participants. Two participants were withdrawn following recruitment and before treatment for receiving IASI from their General Practitioners (GPs) (see Fig. 1); 207 participants participated in the trial and received IASI. A further 8 subjects were later withdrawn for reasons listed in Fig. 1, with 199 participants remaining. Of these, 188 subjects had knee radiographs, mean age was 63.2 years (SD: 10.3 years) and 51.6% were female (see Table 1). Median KOOS-pain score at baseline was 44.4 points (IQR: 36.1–55.6), and median VAS_NA_ was 7.0 cm on a 0–10 cm scale (IQR: 5.6–8.1). For all participants, the median time between baseline and post-injection follow up was 8 days (IQR: 7–14). Of the 120 participants who had MRI, it was possible to assess WORMS in 109 participants and synovial tissue volume in 111 participants.

**Table 1:** Participant characteristics

Variable	MRI cohort	Radiograph cohort
Number	120^a^	188
Age (years), mean (SD)	62.3 (10.3)	63.2 (10.3)
Female, frequency (%)	62 (51.7)	97 (51.6)
Number of days to follow-up appointment, median (IQR)	8.0 (7.0–14.0)	8.0 (7.0–14.0)
KOOS-pain subscale score (0–100)^b^, median (IQR)	44.4 (36.1–55.6)	44.4 (36.1–55.6)
Pain on nominated activity, VAS_NA_ (0–10 cm)^c^, median (IQR)	7.0 (5.5–7.7)^d^	7.0 (5.6–8.1)^e^
Pain in last week, VAS (0–10 cm)^c^, median (IQR)	6.5 (4.7–7.8)^d^	6.5 (4.9–8.0)^e^
Responders to injection, at follow-up visit, frequency, (%)	85 (70.8)	137 (72.9)

*MRI* magnetic resonance imaging, *SD* standard deviation, *IQR* interquartile range, *CI* confidence interval, *KOOS* Knee Injury and Osteoarthritis Outcome Score, *VAS* visual analogue scale, *VAS*_*NA*_ VAS for pain during nominated activity. ^a^There were 109 baseline images available for assessment of Whole Organ Magnetic Resonance Imaging Score and 111 available for assessment of synovial tissue volume; ^b^KOOS pain subscale is scored from 100 (no pain) to 0 (extreme pain); ^c^VAS is scored from 0 cm (no pain) to 10 cm (pain as bad as you can imagine); ^d^there were 5 and 3 patients who did not complete their VAS_NA_ and VAS for pain in the last week, respectively; ^e^there were 8 and 4 patients who did not complete their VAS_NA_ and VAS for pain in last the week, respectively

### Imaging characteristics

Of those with radiographs, 36.7% of participants had overall KL grade 2 at the knee, 53.7% had KL grade 3 and 9.6% had KL grade 4 (see Table 2). For JSN, we considered the scores in the medial tibiofemoral (TF), lateral TF and patellofemoral (PF) compartments and an overall JSN maximal score (any compartment). Overall 1.6% had a JSN maximal score of zero, 31.6% a JSN score of 1, 48.1% a JSN score of 2 and 18.7% a JSN score of 3. Table 2 shows the distribution of the maximal score for the MRI features including BML, meniscal damage, effusion, synovitis, cartilage, osteophytes and attrition.

**Table 2:** MRI and radiographic features at baseline

Maximal score	MRI features (WORMS)							Radiographic features	
	BML (*n*)	Menisci (*n*)	Effusion (*n*)	Synovitis (*n*)	Cartilage (*n*)	Osteophyte (*n*)	Attrition (*n*)	KL^a^ (*n*)	JSN^a^ (*n*)
0	5	35	22	0	5	0	3	0	3
1	30	0	46	2	5	1	19	0	59
2	26	9	32	18	9	10	49	69	90
2.5	-	-	-	-	10	-	-	-	-
3	48	24	9	89	8	19	38	101	35
4		41			26	25		18	
5		0			36	16			
6		0			10	33			
7						5			

*MRI* magnetic resonance imaging, *WORMS* Whole organ magnetic resonance imaging score, *BML* bone marrow lesion, *KL* Kellgren-Lawrence, *JSN* joint space narrowing. WORMS range of scores differs for each feature. ^a^Maximal score in any region; 188 participants with available images for KL grading and 187 for JSN scoring

### Structural parameters and change in pain

At 2 weeks post injection, knee pain significantly improved when assessed by KOOS (+23.7 points; 95% CI: 20.9 to 26.5; *p* < 0.001) and VAS_NA_ (-3.3 cm; 95% CI: -3.8 to -2.9; *p* < 0.001). Among those with MRI scans, those with a higher maximal BML score (b coefficient = 0.76, 95% CI 0.16 to 1.36) and maximal meniscal damage score (b coefficient = 0.37, 95% CI 0.04 to 0.70) had less improvement in pain as determined by VAS_NA_ (see Table 3). Similarly, among those with radiographs, higher TF and PF KL scores, higher overall KL maximal score, higher PF JSN score and higher overall JSN maximal score were associated with less improvement in pain as assessed using VAS_NA_ (see Table 3). The presence of synovitis assessed using WORMS and also STV assessed quantitatively was not associated with change in pain. For pain assessed using KOOS, the results in relation to structural predictors were in the same direction though they were not statistically significant. Mal-alignment was not associated with response.

**Table 3:** Baseline imaging characteristics and their relation to change in pain at 2 weeks among persons treated with intra-articular steroids

Predictor variable in regression	KOOS pain score			Pain on nominated activity VAS		
	*N*	b coefficient (95% CI)^c^	*P* value	*N*	b coefficient (95% CI)^d^	*P* value
MRI (WORMS)						
Synovitis maximal score (0–3)	109	-1.94 (-11.15 to 7.26)	0.677	96	0.45 (-0.83 to 1.74)	0.487
Bone marrow lesion maximal score (0–3)	109	-3.48 (-7.76 to 0.80)	0.110	96	0.76 (0.16 to 1.36)*	0.013
Bone attrition maximal score (0–3)	109	-1.78 (-6.98 to 3.42)	0.498	96	-0.07 (-0.81 to 0.66)	0.846
Cartilage damage maximal score (0–6)	109	0.09 (-2.55 to 2.72)	0.948	96	0.16 (-0.20 to 0.53)	0.378
Cyst maximal score (0–3)	108	0.56 (-4.48 to 5.60)	0.827	95	0.27 (-0.48 to 1.01)	0.475
Menisci damage maximal score (0–6)	109	-2.12 (-4.49 to 0.25)	0.079	96	0.37 (0.04 to 0.70)*	0.027
Osteophyte maximal score (0–7)	109	0.56 (-2.23 to 3.36)	0.690	96	0.18 (-0.22 to 0.57)	0.377
Synovial effusion (0–3)	109	-1.22 (-5.90, 3.46)	0.608	96	0.17 (-0.49 to 0.83)	0.606
MRI (quantitative)						
Synovial tissue volume (1000 mm^3^)	111	-0.05 (-0.67 to 0.57)	0.883	98	0.03 (-0.05 to 0.12)	0.408
Radiographs						
Tibiofemoral KL score (0–4)	186	-2.40 (-6.80 to 2.00)	0.284	170	0.66 (0.02 to 1.30)*	0.042
Patellofemoral KL score (0–4)	187	-0.001 (-3.16 to 3.16)	0.999	171	0.64 (0.18 to 1.10)*	0.006
Overall KL maximal score^a^ (0–4)	187	-1.97 (-6.64 to 2.71)	0.408	171	0.94 (0.27 to 1.62)*	0.007
Lateral tibiofemoral JSN score (0–3)	185	-0.001 (-4.12 to 4.12)	1.000	169	0.05 (-0.56 to 0.67)	0.865
Medial tibiofemoral JSN score (0–3)	186	-1.26 (-5.17 to 2.65)	0.524	170	0.21 (-0.38 to 0.79)	0.486
Patellofemoral JSN score (0–3)	178	-0.52 (-3.60 to 2.56)	0.738	162	0.52 (0.06 to 0.98)*	0.026
Overall JSN maximal score^b^ (0–3)	186	-1.34 (-5.26 to 2.57)	0.499	170	0.72 (0.14 to 1.29)*	0.015
Knee mal-alignment angle (per degree)	191	0.03 (-0.53 to 0.59)	0.924	173	-0.04 (-0.13 to 0.04)	0.291

*KOOS* Knee Injury and Osteoarthritis Outcome Score, *VAS* visual analogue scale, *MRI* magnetic resonance imaging, *WORMS* Whole Organ Magnetic Resonance Imaging Score, *KL* Kellgren-Lawrence, *JSN* joint space narrowing. Results shown are from fixed panel regression. ^a^maximal KL grade of the tibiofemoral or patellofemoral joint; ^b^maximal JSN score of the lateral or medial tibiofemoral or patellofemoral joint; ^c^KOOS pain score ranges from 0–100 with lower scores representing more severe pain. Negative coefficients signify that more severe imaging findings were associated with less pain improvement; ^d^pain on nominated activity ranged from 0 to 10 with higher values representing more severe pain. Positive coefficients signify that more severe imaging findings were associated with less pain improvement. *Represent results that are significant at *p* < 0.05

### Predictors of responder status

#### Short term response

At short term follow up 146 (73.4%) of the 199 participants were OMERACT-OARSI responders. None of the MRI or radiographic variables was associated with OMERACT-OARSI responder status at short term follow up (see Table 4).

**Table 4:** Prediction of responder status at short term (2 weeks) and longer term (6 months) follow up, by baseline imaging characteristics

Predictor variable in regression	Short term responder at 2 weeks (yes/no)			Longer term responder at 6 months (yes/no)		
	*N*	Odds ratio (95% CI)	*P* value	*N*	Odds ratio (95% CI)	*P* value
MRI (WORMS)						
Synovitis maximal score (0–3)	109	0.82 (0.32 to 2.13)	0.689	109	0.43 (0.17 to 1.12)	0.084
Bone marrow lesion maximal score (0–3)	109	0.76 (0.49 to 1.19)	0.231	109	1.12 (0.66 to 1.92)	0.668
Bone attrition maximal score (0–3)	109	0.94 (0.56 to 1.57)	0.804	109	0.59 (0.32 to 1.10)	0.097
Cartilage damage maximal score (0–6)	109	0.98 (0.75 to 1.27)	0.869	109	1.03 (0.74 to 1.42)	0.873
Cyst maximal score (0–3)	108	0.74 (0.46 to 1.20)	0.222	108	1.27 (0.72 to 2.22)	0.408
Menisci damage maximal score (0–6)	109	0.95 (0.74 to 1.20)	0.647	109	0.74 (0.55 to 0.98)*	0.039
Osteophyte maximal score (0–7)	109	1.13 (0.86 to 1.49)	0.386	109	0.98 (0.70 to 1.38)	0.919
Synovial effusion (0–3)	109	0.83 (0.52 to 1.31)	0.420	109	0.79 (0.44 to 1.41)	0.421
MRI (quantitative)						
Synovial tissue volume (per 1000 mm^3^)	111	1.00 (1.00 to 1.00)	0.612	111	0.96 (0.88 to 1.04)	0.327
Radiographs						
Tibiofemoral KL score (0–4)	187	0.79 (0.49 to 1.28)	0.341	187	0.52 (0.30 to 0.91)*	0.021
Patellofemoral KL score (0–4)	188	0.86 (0.60 to 1.22)	0.391	188	0.80 (0.54 to 1.19)	0.269
Overall KL maximal score^a^ (0–4)	188	0.72 (0.43 to 1.20)	0.205	188	0.43 (0.23 to 0.82)*	0.010
Lateral tibiofemoral JSN score (0–3)	186	1.27 (0.79 to 2.03)	0.327	186	0.86 (0.51 to 1.45)	0.569
Medial tibiofemoral JSN score (0–3)	187	0.91 (0.59 to 1.40)	0.661	187	0.69 (0.42 to 1.15)	0.154
Patellofemoral JSN score (0–3)	179	0.83 (0.59 to 1.16)	0.265	179	0.85 (0.56 to 1.28)	0.425
Overall JSN maximal score^b^ (0–3)	187	0.75 (0.49 to 1.16)	0.196	187	0.60 (0.36 to 0.99)*	0.047
Knee mal-alignment angle (per degree)	192	0.98 (0.92 to 1.04)	0.481	192	1.01 (0.94 to 1.08)	0.801

*MRI* magnetic resonance imaging, *WORMS* Whole Organ Magnetic Resonance Imaging Score, *KL* Kellgren-Lawrence, *JSN* joint space narrowing. Results presented are from logistic regression. ^a^maximal KL grade of the tibiofemoral or patellofemoral joint; ^b^maximal JSN score of the lateral or medial tibiofemoral or patellofemoral joint. *Represent results that are significant at *p* < 0.05

#### Longer term response

Of the 146 participants who were short term responders, 6 did not attend for a final visit. Of those who did, 100 had a recurrence of pain during the following 6 months, while 40 were longer term responders. Higher maximal score for meniscal damage, and higher radiographic TF KL score, overall KL maximal score and overall JSN maximal score were associated with decreased odds of being a longer term responder (see Table 4).

### Predicted probability of response

Depending on the structural predictor, as disease severity increased there was a trend towards a reduced probability of being a responder to IASI. For instance, the probability of being a short term responder to IASI decreased from 82% (95% CI 69% to 95%) among those with an overall JSN maximal score of 0, to 66% (95% CI 53% to 79%) among those with a JSN maximal score of 3, though this difference was not statistically significant (see Table 5). In the longer term there was a significant threefold decrease in the chance of being a longer term responder to IASI when the overall JSN maximal scores increased from 0 (38%; 95% CI 16% to 60%) to 3 (12%; 95% CI 4% to 19%) and a fivefold decrease when the maximal meniscal score increased from 0 (28%; 95% CI 14% to 43%) to 6 (6%; 95% CI 0% to 13%).

**Table 5:** Predictive probabilities of being a short term and longer term responder: radiographs and MRI features

	Probability						
Overall KL maximal grade^a^	0	1	2	3	4		
Short term predicted probability^b^	0.87^d^	0.83^d^	0.78	0.71	0.64		
Longer term predicted probability	0.69^d^	0.49^d^	0.29	0.15	0.07		
Overall JSN maximal score^c^	0	1	2	3			
Short term predicted probability^b^	0.82	0.77	0.72	0.66			
Longer term predicted probability	0.38	0.27	0.18	0.12			
Menisci maximal WORMS	0	1	2	3	4	5	6
Short term predicted probability	0.72	0.70	0.69	0.68	0.67	0.66	0.64
Longer term predicted probability	0.28	0.22	0.18	0.14	0.10	0.08	0.06

*KL* Kellgren-Lawrence, *JSN* joint space narrowing, *WORMS* Whole Organ Magnetic Resonance Imaging Score; ^a^maximal KL grade of the tibiofemoral or patellofemoral joint; ^b^trend not statistically significant; ^c^maximal JSN score of the lateral or medial tibiofemoral or patellofemoral joint; ^d^values based on modelling

## Discussion

In this study, 73.4% of participants were short term responders after an IASI, while one in four was a longer term responder. Using OMERACT-OARSI criteria as our definition of response, we found no short term structural predictors of response. Increasing disease severity as determined by MRI and plain radiographs was associated with longer term non-responder status.

Radiographic markers of disease severity including higher KL and JSN scores were associated with poorer response to IASI in the short term as defined by a change in VAS_NA_. Similarly, MRI features were also linked with response, with features suggestive of more structural damage being linked with a poorer short term response.

There are few studies that have looked at the influence of structural factors on response to IASI. In a systematic review we found a paucity of data on predictors of response to IASI and inconsistent results relating to disease severity [8]. The findings of the current study are consistent with two studies where increasing radiographic severity of OA was linked with reduced response [7, 15]. Smith et al. [7] in a study of 38 patients with knee OA who received 120 mg methylprednisolone acetate preceded by arthroscopy, found that severity was linked with reduced OARSI response at 4 weeks, while in a study of 79 participants with OA, higher radiographic grade was linked with less improvement in Western Ontario and McMaster Universities Osteoarthritis Index (WOMAC) pain score at 26 weeks (*p* < 0.05); however, the analysis was based on combined outcome of their steroid and lavage/tidal irrigation groups [15]. In contrast, two other studies did not find that radiographic OA grading predicted short term response (<6 weeks) to IASI in knee OA when VAS pain was used as the outcome [9, 13]. The small sample size of these studies (16–42 participants) might have contributed to low power to detect response [9, 13]. Another factor, which may have explained the potential discrepancy, is the dose of steroid used. The two previous positive studies used either 40 mg of triamcinolone acetonide or 120 mg methylprednisolone acetate [7, 15], while the two that did not show any association of severity of disease with response used a smaller dose - 20 mg triamcinolone hexacetonide [9, 13]. Further, the focus of prior studies on short term outcomes may have also made it difficult for these studies to detect an association between disease severity and outcome, as the large majority of patients respond over the short term. Additionally no previous studies have examined both short term and longer term predictors of outcomes within the same trial. In our study we found no significant associations between structural damage and short term response as defined by a change in KOOS; however, the direction of effect (suggesting a poorer response) was similar to that observed for the VAS_NA_.

To our knowledge, this is also the first study to have looked at MRI predictors of response. The results are consistent with the radiographic findings, though perhaps because of smaller numbers in the responder analysis, not all MRI features attained statistical significance. The association between higher meniscal damage with a lesser reduction in pain in the short term, and also reduced odds of being a longer term responder, is consistent with greater meniscus damage being associated with greater severity of OA disease [24]. Larger BMLs were also associated with a lesser reduction in pain in the short term; previous studies have shown that increase in the size of BMLs is associated with increased severity of joint degeneration [25–27].

At the outset, we hypothesised that there would be a link between synovitis and response, with those with greater baseline synovitis being more likely to respond; however, we did not find this in the study. The mechanism of the therapeutic effect of corticosteroids in knee OA is unclear, though it has been thought likely related in part to their potent anti-inflammatory effect. We have already shown correlation between change in STV, which is a marker for synovitis, and change in pain, in a previous paper [16] and had expected to find a link between baseline synovitis and response. The most plausible explanation for the apparent discordant findings is that any within-person change in pain due to reduced synovitis is masked by other sources of between-person variations in levels of pain including structural factors. The null findings, though, are in keeping with observations made in previous studies that used either clinical or ultrasound assessment of synovial hypertrophy and power Doppler assessment of synovitis [6, 10, 11].

The presence of an effusion on MRI was not a predictor of response. Previous studies that correlated positive response to IASI with effusion had used clinical assessment to detect knee effusion [9, 15], though one other study that used clinical assessment of effusion found no such correlation [10]. Non-contrast ultrasound assessment of effusion has not been shown to predict response [6, 11].

In our study, the early-responder rate (73.4%) was similar to most though not all studies [1]. There are much fewer data on longer term response. We found 20% of participants maintained their improvement in pain for at least 6 months. This is consistent with Arden et al. [15] who reported that 29% of participants maintained improvement at their 6-month follow up. Smith et al. [7] reported a higher (42% or 16 participants) longer term responder rate at 6 months; it is possible this could be explained by the higher dose of steroid used (120 mg methylprednisolone acetate) and also by the fact that 30 out of their 38 participants received a second course of IASI at the time of relapse during the period of the study.

Using radiographic and MRI data, we derived a predictive table, though the short term radiographic prediction probabilities should be viewed with caution given their non-significant results and their wide CIs. Our predictive probabilities suggest people with advanced radiographic disease are unlikely to benefit from IASI in the longer term. Based on the degree of JSN observed on radiographs, the odds of having longer term response is increased from around 10% to almost 40% as the disease becomes less severe.

Our study is the largest yet performed looking at response to IASI. There are some limitations to be considered in interpreting the analysis. The study included individuals who received steroid injections only and it is likely that the overall response to the injection is at least in part due to a “placebo” effect. It seems, however, very unlikely that this can explain our findings relating to factors identified as predicting response. As noted previously, earlier studies of intra-articular steroids without a control group have not reported an association between structural severity and response, and while structural severity has been linked to a worse pain trajectory in some studies [28], other longitudinal studies have reported no association between baseline structural severity and the trajectory of pain [29, 30]. In relation to limb mal-alignment we used conventional non-standardised images to estimate the angle and did not use a full-limb weight-bearing radiograph, which is a gold standard for measuring knee mal-alignment; it is possible that misclassification may have resulted in a bias of the results towards the null. There is, however, evidence of strong correlation between the femur-tibia angle measured from knee radiographs with hip-knee-ankle angle measured from full-limb radiographs (*r* = 0.86; 95% CI: 0.81 to 0.90) [23].

Our MRI sequences were optimised for the detection of BMLs, which may have contributed to our negative findings with the other knee structures. Further, radiographs in the study could have been taken up to 24 months prior to the IASI and it is possible that progression of disease may have led to some misclassification of radiographic disease severity with underestimation of OA severity in a small proportion of subjects. Based on data from the MOST study we estimate that over a 24-month period up to 20% of subjects would have KL progression; however, for most subjects in our study the time interval was significantly shorter [31]. It is possible, however, that this may have had a small impact on the predictive probabilities observed. Some subjects did not complete the pain questions at baseline and follow up. We have repeated the analysis using complete case analysis based on data from participants with complete data on all variables with similar results to the maximal case analysis.

## Conclusion

Over two thirds of people with painful knee OA respond significantly in the short term to IASI and one in four have persisting benefit beyond 6 months. Those with more severe knee joint damage are less likely to respond to knee IASI in the longer term. In contrast, the presence of synovitis/effusion does not appear to predict successful response to steroid injection therapy.

## Abbreviations

AP: Antero-posterior
BML: Bone marrow lesion
CE: Contrast-enhanced
GP: General Practitioner
IASI: Intra-articular steroid injection
ICC: Intra-class correlation coefficient
IQR: Interquartile range
JSN: Joint space narrowing
KL: Kellgren-Lawrence
KOOS: Knee injury and osteoarthritis outcome score
MRI: Magnetic resonance imaging
MSK: musculoskeletal
OA: Osteoarthritis
OARSI: Osteoarthritis Research Society International
OMERACT: Outcome Measures in Rheumatology
OR: Odds ratio
PF: Patellofemoral
STV: Synovial tissue volume
TF: Tibiofemoral
VAS: Visual analogue scale
VAS_NA_: Visual analogue scale for pain during nominated activity
WORMS: Whole organ magnetic resonance imaging score
